# Bilateral dermoid ovarian torsion in a young woman: a case report

**DOI:** 10.1186/s13256-018-1698-8

**Published:** 2018-06-08

**Authors:** Saeed Baradwan, Wed Sendy, Sameer Sendy

**Affiliations:** 1 Department of Obstetrics and Gynecology, Women’s Specialized Hospital, King Fahad Medical City, Riyadh, Saudi Arabia; 2 Faculty of Medicine, Princess Nourah Bint Abdulrahman University, Riyadh, Saudi Arabia

**Keywords:** Ovarian torsion, Laparoscopy, Adolescent, Dermoid, Ovarian detorsion

## Abstract

**Background:**

Of all patients undergoing emergency surgery for acute pelvic pain, approximately 2.7% of cases are caused by ovarian torsion. We report a rare occurrence of bilateral ovarian torsion in a young woman.

**Case presentation:**

We report the case of a 20-year-old white woman who presented with sudden onset of severe lower abdominal pain and nausea. Similar episodes of pain were experienced in the previous few months and diagnosed as a case of bilateral ovarian cyst. She was found to have a bilateral ovarian torsion caused by adnexal mass. She was treated by laparoscopic detorsion, left salpingo-oophorectomy, and right cystectomy.

**Conclusion:**

This case highlights the need to perform an early laparoscopic surgical intervention in cases of bilateral ovarian mass because of the greater chance for their torsion and subsequent effects on fertility.

## Background

Of all patients undergoing emergency surgery for acute pelvic pain, approximately 2.7% of cases are caused by ovarian torsion [1]. It is defined as the partial or complete rotation of adnexa around its vascular axis that may cause an interruption in the ovarian blood and lymphatic flow [2]. It is reported to occur at any age from pre-puberty to post-menopause with the greatest incidence in women 20–30 years of age [3]. The most common benign tumor reported to have ovarian torsion is dermoid and its diagnosis is based on the clinical presentation. If ovarian torsion is suspected, then an emergency surgical intervention should be performed to prevent ovarian damage.

We report a rare case of bilateral ovarian torsion treated by laparoscopic unilateral oophorectomy, ovarian detorsion, and cystectomy on the other side with the aim of preserving her fertility.

## Case presentation

A 20-year-old white woman, para 1 + 0, presented to our institution with a history of sudden onset of severe lower abdominal pain and nausea. The pain was described as constant, sharp, radiated to her back, and associated with episodes of vomiting after a few hours. Similar episodes of pain were experienced in the previous few months and diagnosed as a case of bilateral dermoid in another hospital but the case was not documented. These episodes of pain were, however, shorter in duration and resolved spontaneously. She was otherwise well and there were no other associated gastrointestinal or genitourinary symptoms. She had no previous history of any illnesses or allergies. She denied the use of any medications. She had one pregnancy, usual course, and delivered normal spontaneous vaginal delivery. There was no family history of malignancies. There was no significant family or psychosocial history. Her menarche commenced at the age of 11 years with subsequent regular cycles.

On physical examination, she was alert, in mild distress, and her vital signs were within normal limits. An abdominal examination showed lower abdominal tenderness, with muscle guarding to palpation but there was no distension. Intestinal sounds were normal. An external genital examination was normal. A pelvic examination revealed bilateral adnexal tenderness on vaginal touch. Her full blood counts, serum biochemistry, and tumor marker were within the normal ranges. Urine analysis and pregnancy test were negative.

An ultrasound examination was performed bedside demonstrating bilateral ovarian cystic masses, on the left side around 14 × 11 cm and right side 8 × 6 cm with negative Doppler flow in the ovarian tissue with evidence of solid components. On the basis of these findings, ovarian torsion caused by adnexal mass was the likely diagnosis. Our patient was counselled and signed informed consent for laparoscopic ovarian detorsion, cystectomy, possible oophorectomy, and laparotomy if needed.

**Fig. 1:**
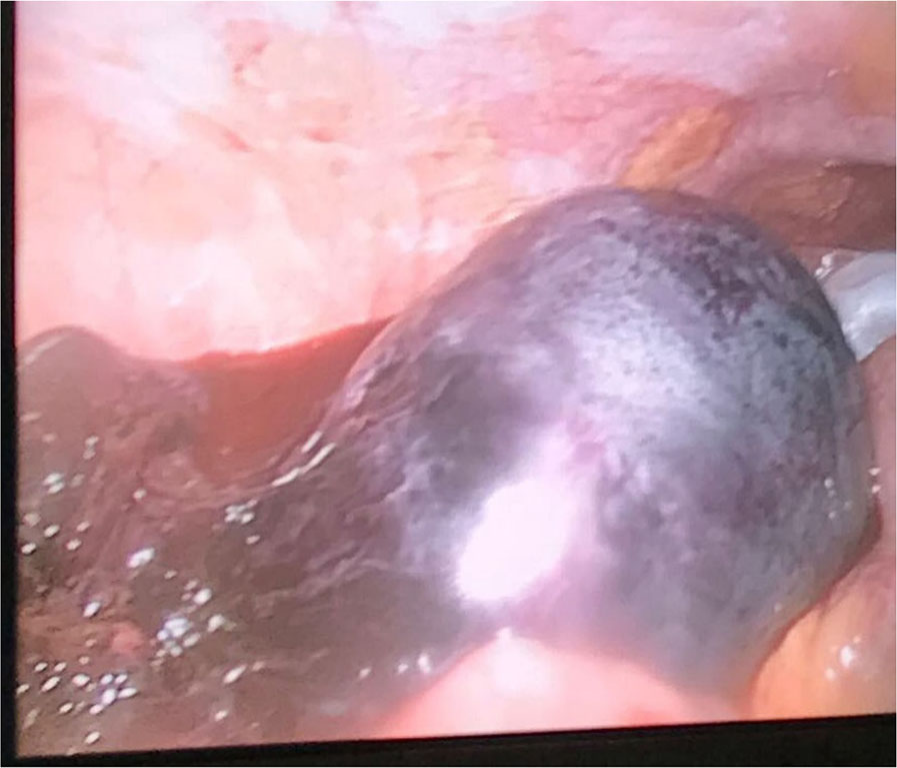
Intraoperative findings: the left ovary looked necrotic and was found to be twisted twice over

**Fig. 2:**
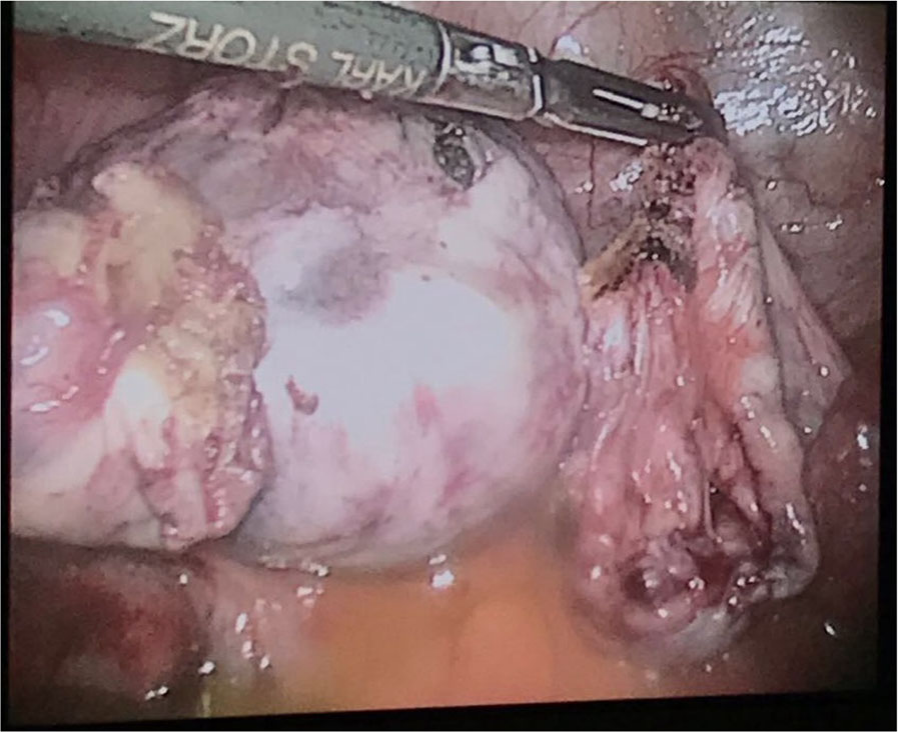
Intraoperative findings: the right ovary improved in color after detorsion

Intraoperative findings confirmed a bilateral ovarian torsion that was the same size as that found by ultrasound. Our patient’s left ovary looked necrotic and was found to be twisted twice over. Detorsion of both ovaries were performed but the necrotic appearance of her left ovary did not improve and bleeding was observed from the necrotic ovary (Fig. 1) but her right ovary improved in color (Fig. 2). The decision was made by the surgical team to perform a left-sided salpingo-oophorectomy and right cystectomy. There were no major intraoperative complications. The pain resolved completely after surgery and the final pathologic diagnosis was mature ovarian teratoma (dermoid). She was discharged on the second postoperative day and advised to follow up after 4 weeks. Her postoperative period was uneventful. She recovered completely from her surgery and has gone back to her normal daily activity.

## Discussion

An ovarian dermoid cyst (benign cystic teratoma) is a benign tumor originating from germinal cells. On histologic examination, they are composed of variable proportions of tissue originating from the ectoderm, mesoderm, and endoderm [4]. It has been reported that this lesion occurs in women aged between 20 and 30 years in approximately 80% of cases and represents 18 to 20% of benign ovarian tumors. Usually, dermoid cysts are unilateral, but they are bilateral in 10 to 15% of cases [5, 6]. The present case is 20 years of age and presented with bilateral dermoid cysts. Acute abdominal and pelvic pain is the commonest symptom of a dermoid cyst, and, in 15% of cases, the symptoms are associated with menstrual abnormalities. The commonest complication is torsion, whereas rupture and suppuration are uncommon complications [7]. In the present case, acute sharp and constant pelvic pain was the presenting symptom and bilateral dermoid cyst torsion was diagnosed.

Imaging studies, including high-resolution real-time two-dimensional ultrasonography and computed tomography, are helpful in the differential diagnosis of dermoid cysts, but are not so useful in showing the site of torsion of the ovarian cysts. Color flow Doppler mapping may be helpful in localizing the tumor torsion [8]. In the present case, the diagnosis was confirmed by a bedside ultrasound examination which demonstrated bilateral ovarian cystic masses, accompanied by negative Doppler flow in the ovarian tissue with evidence of solid components.

Surgical intervention is indicated if torsion, rupture, or hemorrhage of dermoid cysts is expected [9, 10]. The present case of bilateral dermoid cysts torsion was treated by laparoscopic unilateral oophorectomy, ovarian detorsion, and cystectomy on the other side. The main aim was to preserve her fertility.

After discussing the risks and benefits with our patient, we decided to perform diagnostic laparoscopy. Previous authors have reported that laparoscopic removal of ovarian dermoid cysts is a safe procedure [11, 12].

The main issue in this case as a retrospective case, was that she was diagnosed as a case of bilateral ovarian mass a few months back and no interventions or follow up were obtained. At the end, her cyst was complicated by torsion and she lost one ovary. Our recommendation from this case is early laparoscopic surgical intervention in cases of bilateral ovarian mass to prevent their complications.

The benefits of performing laparoscopy are numerous and include lower risk of wound complications, less postoperative pain and ileus, shorter hospital stays, reduced adhesion formation, and a faster return to normal activities [13].

## Conclusion

We have reported a rare case of bilateral dermoid ovarian torsion in a young woman and highlight the need to perform an early laparoscopic surgical intervention in cases of bilateral ovarian mass because of the greater chance for their torsion and subsequent effects on fertility.
